# Diagnostic and prognostic value of ventilatory power in pulmonary hypertension

**DOI:** 10.1186/s12931-022-02212-5

**Published:** 2022-10-16

**Authors:** Xin Li, Yi Zhang, Qi Jin, Zhihui Zhao, Qing Zhao, Lu Yan, Anqi Duan, Zhihua Huang, Meixi Hu, Changming Xiong, Qin Luo, Zhihong Liu

**Affiliations:** 1 Center for Pulmonary Vascular Diseases, National Center for Cardiovascular Diseases, Chinese Academy of Medical Sciences and Peking Union Medical College, Fuwai Hospital, No.167 Beilishi Rd, Xicheng District, Beijing, 100037 China; 2 Department of Cardiology, Zhongshan Hospital, Shanghai Institute of Cardiovascular Diseases, Fudan University, Shanghai, China

**Keywords:** Pulmonary hypertension, Ventilatory power, Severity, Prognosis

## Abstract

**Background:**

Ventilatory power is a novel index which could reflect both ventilation efficiency and peripheral blood flow. However, its clinical value in pulmonary hypertension (PH) is rarely discussed. In the present study, we aimed to investigate the diagnostic and prognostic value of ventilatory power as well as its association with disease severity in PH.

**Methods:**

Consecutive patients with normal hemodynamics and patients diagnosed with PH between September, 2012 and December, 2020 in Fuwai hospital were enrolled. Receiver operating characteristic curves were constructed to determine diagnostic power of ventilatory power and tricuspid regurgitation velocity (TRV). Spearman correlation coefficients were used to evaluate bivariate correlation. Multivariable Cox analysis were used to evaluate the association between ventilatory power and clinical worsening.

**Results:**

A total of 679 patients were included in the study, among whom 177 were patients with normal hemodynamics, and 502 were patients with PH. Among patients with PH, those experiencing clinical worsening had lower ventilatory power than those did not. The area under the curve of TRV plus ventilatory power was higher than TRV used alone when identifying overt and borderline PH. Ventilatory power was also correlated with well-validated variables that reflected severity of PH, such as NT-proBNP. Multivariable Cox analysis showed that ventilatory power could independently predict clinical worsening and could improve the predictive power of the current PH risk assessment tool.

**Conclusion:**

Ventilatory power could improve the predictive power of TRV in identifying overt PH and borderline PH. Moreover, it could reflect disease severity and independently predict clinical worsening.

**Supplementary Information:**

The online version contains supplementary material available at 10.1186/s12931-022-02212-5.

## Introduction

Exercise capacity is of great value in the management of patients with pulmonary hypertension (PH). Cardiopulmonary exercise test (CPET) serves as the gold standard in evaluating exercise capacity due to its objectiveness and accuracy [1]. Previous studies have confirmed that oxygen consumption at peak exercise (VO_2_@Peak) and minute ventilation/ carbon dioxide production (VE/VCO_2_) slope could independently predict survival of patients with PH and were applied for risk stratification [2]. However, VO_2_@Peak focused exclusively on cardiac-derived blood flow and could not reflect peripheral perfusion. Similarly, VE/VCO_2_ slope only illustrated ventilatory response to increased pulmonary perfusion during exercise but failed to take peripheral blood pressure into consideration.

Ventilatory power, calculated by systolic blood pressure at peak exercise (SBP@Peak)/ VE/VCO_2_ slope, is a novel index which could reflect both ventilation efficiency and peripheral blood flow [3]. Nevertheless, the literature on the clinical value of ventilatory power is scarce. Forman et al. [4] firstly reported that ventilatory power could independently predict survival in patients with left heart failure, and demonstrated superior predictive ability to VO_2_@Peak and VE/VCO_2_ slope. However, the prognostic value of ventilatory power in patients with PH characterized by right heart failure remains unknown. Correale et al. [5] reported that ventilatory power was associated with hemodynamics in patients with PH and could differentiate patients with PH from those with mean pulmonary arterial pressure (mPAP) < 25 mmHg. Unfortunately, their sample size was relatively small (n = 47) and they did not investigate the diagnostic performance in differentiating patients with borderline PH (mPAP = 21–25 mmHg) from those with mPAP ≤ 20 mmHg.

The aim of current study is to evaluate diagnostic and prognostic value of ventilatory power as well as its association with disease severity in patients with PH.

## Methods

### Study design and population

This retrospective cohort study was conducted in Fuwai hospital, Chinese Academy of Medical Sciences (Beijing, China). Consecutive echocardiography-suspected PH patients with invasively measured mPAP < 25 mmHg, and both incident and prevalent patients diagnosed with chronic thromboembolic pulmonary hypertension (CTEPH) or idiopathic pulmonary arterial hypertension (IPAH) between September, 2012 and December, 2020 were screened. The establishment of CTEPH and IPAH was in accordance with the European Society of Cardiology (ESC)/European Respiratory Society (ERS) guidelines [2, 6]. Inclusion criteria were: (1) patients underwent right heart catheterization (RHC); (2) patients underwent CPET; (3) the time interval between CPET and RHC was less than 3 months. Exclusion criteria were: (1) patients with pulmonary diseases and mPAP ≤ 40 mmHg (the mPAP of pure group 3 PH usually does not exceed 40 mmHg) [7, 8]; (2) incomplete CPET data for calculating ventilatory power at baseline; (3) loss to follow-up (except for patients with mPAP < 25 mmHg).

Demographic, echocardiographic, RHC-derived, and CPET-derived parameters were collected from an electronic medical record system by two independent reviewers (XL and QJ).

### Risk stratification strategy

We used the abbreviated 2015 ESC/ERS risk stratification strategy to categorize patients as low, intermediate or high risk [9]. One to three points were assigned to each parameter in this prediction model, which included World Health Organization Function Class (WHO FC), six-minute walk distance (6MWD), N-terminal pro-B-type natriuretic peptide (NT-proBNP), right arterial pressure, cardiac index, and mixed venous oxygen saturation (S_v_O_2_) (details are shown in Additional file 2: Table S1). The risk score for each patient was calculated as follows: the sum of all points / the number of variables available (rounding decimal to the nearest integer).

### Cardiopulmonary exercise test

CPET was performed as described previously [10]. Initially, patients rested on an upright cycle ergometer (COSMED, Rome, Italy) for 3 min and then pedaled without workload for another 3 min. Subsequently, work rate increased gradually (5 to 30 W/min) in accordance with individual’s estimated exercise tolerance until exhaustion or symptom limitation. Anaerobic threshold was identified by ventilation equivalents method. SBP@Peak was considered the highest SBP value achieved during the CPET. VE/VCO_2_ slope (VE plotted versus VCO_2_ slope) was calculated by linear regression from rest to peak exercise. Ventilatory power was calculated as the ratio of SBP@Peak to VE/VCO_2_ slope [11].

### Follow-up

After discharge, patients were followed up regularly via outpatient/inpatient examination or telephone call until the first occurrence of the outcome or end of the study, namely 1st November, 2021.

### Outcome

Clinical worsening was the primary outcome for this study, which was defined as the first occurrence of any of the following events: all-cause death, lung transplant, rehospitalization due to heart failure/deterioration of PH and addition of parenteral prostanoids. Time to clinical worsening was calculated by the time interval between the date of CPET and the end of follow-up. All possible events were audited independently by two senior clinicians. Upon discordance, consensus was achieved by the supervisors through discussion (QL and ZHL).

### Statistical analysis

Kolmogorov-Smirnova test was performed to test data distribution. Normally distributed continuous parameters were presented as the mean ± standard deviation and compared by independent-sample *t* test, while abnormally distributed continuous parameters were presented as the median (interquartile range) and compared by Mann–Whitney *U* test. Categorical variables were presented as counts (percentages) and the Chi-square test with or without continuity correction or Fisher exact test was used to compare difference between groups. Spearman correlation coefficients were used to evaluate bivariate correlation. One-way analysis of variance was used to compare difference among different risk strata with LSD post hoc test. To evaluate the diagnostic performance of ventilatory power, all included patients with mPAP < 25 mmHg (n = 177) were matched 1:1 with those with overt PH (mPAP ≥ 25 mmHg) by age (± 3) and gender (using SPSS 25.0). To evaluate the diagnostic performance of ventilatory power in identifying borderline PH, all included patients with mPAP = 21–24 mmHg (n = 34) were matched 1:1 with those with mPAP ≤ 20 mmHg by age (± 3 years) and gender (using SPSS 25.0). Receiver operating characteristic (ROC) curves were constructed to determine diagnostic power of ventilatory power and tricuspid regurgitation velocity (TRV). DeLong’s test was used to compare the area under curve (AUC) under ROC curves. Linearity between variables and clinical worsening was examined by restricted cubic splines with four knots. Then, univariable Cox proportional hazards models were applied to identify prognostic factors associated with clinical worsening. Subsequently, parameters of *P* < 0.100 in univariable model were included into multivariable Cox model with forward stepwise method (Likelihood Ratio). The Kaplan–Meier method and the log-rank test were used to analyze the difference in Kaplan–Meier curves between patients with above or below the median value of ventilatory power. The significance was considered at *P* value less than 0.05. Statistical analysis was performed with SPSS 25.0 (IBM SPSS Corp.; Armonk, NY, USA), GraphPad Prism 8.0 (GraphPad Software, Inc.; San Diego, CA, USA) and R (version 4.0.5, R Foundation for Statistical Computing, Vienna, Austria).

## Results

### Patients enrollment

**Fig. 1:**
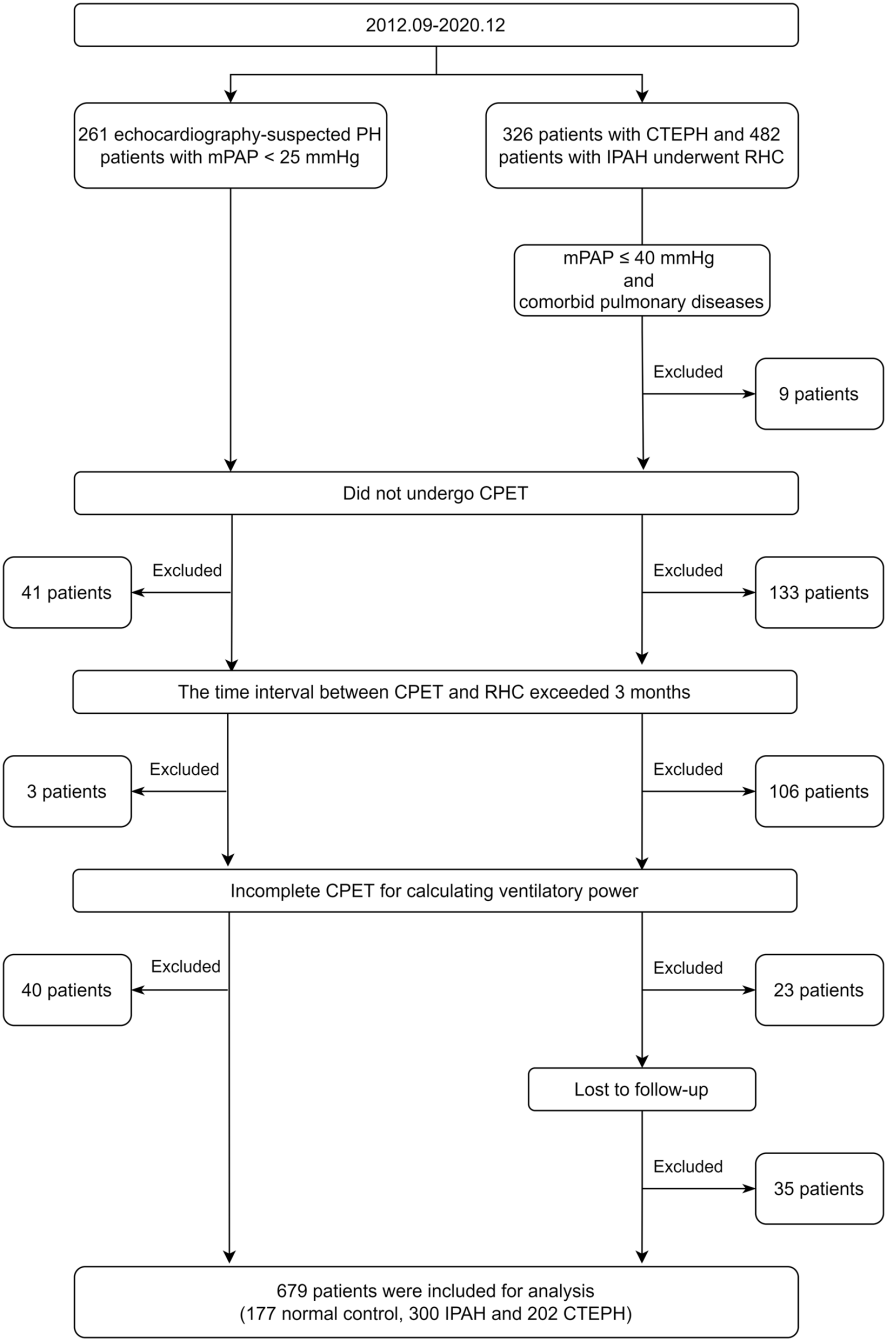
Flow Diagram of Participant Enrollment and Exclusion. CPET, cardiopulmonary exercise testing; CTEPH, chronic thromboembolic pulmonary hypertension; IPAH, idiopathic pulmonary arterial hypertension; mPAP, mean pulmonary arterial pressure; PH, pulmonary hypertension; RHC, right heart catheterization

From September, 2012 to December, 2020, 261 echocardiography-suspected PH patients with mPAP < 25 mmHg, 326 patients with CTEPH, and 482 patients with IPAH underwent RHC in Fuwai hospital. Among them, 390 patients were excluded: (1) patients with pulmonary diseases and mPAP ≤ 40 mmHg (n = 9); (2) not undergoing CPET (n = 174); (3) the time interval between CPET and RHC exceeded 3 months (n = 109); (4) incomplete CPET for calculating ventilatory power (n = 63); (5) loss to follow-up (n = 35). Finally, a total of 679 patients were included into the study (177 with mPAP < 25 mmHg, 300 with IPAH and 202 with CTEPH) (Fig. 1). Among patients with IPAH and CTEPH, 66.1% were female and the median follow-up period was 664 days (interquartile range: 263, 1148). During the follow-up, 192 patients experienced clinical worsening. More specifically, 17 patients died, 146 patients rehospitalized for heart failure or deterioration of PH, and 29 patients received additional parenteral prostanoids.

### Baseline characteristics

Tables 1 and 2 summarize the baseline features of all included patients. In PH group, patients with and without clinical worsening were comparable in terms of age, body mass index, sex, etiology of PH, and PH treatment. However, patients with clinical worsening had worse cardiac function, as reflected by worse WHO FC, higher NT-proBNP, larger ratio of right ventricular end-diastolic diameter/ left ventricular end-diastolic diameter (RVED/LVED), lower cardiac index, and higher PVR. Moreover, exercise capacity was also poorer in these patients, as illustrated by shorter 6MWD, lower VO_2_@Peak, and lower ventilatory power [median (interquartile range): 2.73 (2.08, 3.58) mmHg vs. 3.12 (2.33, 4.04) mmHg, *P* = 0.002].

**Table 1:** Demographic Features of Included Patients

Variables	Normal control (n = 177)	PH			
		Total (n = 502)	Non-CW (n = 310)	CW (n = 192)	*P* value^#^
Age, years	49 (37, 58)	39 (29, 54)**	41 (29, 54)	37 (29, 55)	0.541
Body mass index, kg/m^2^	23.2 (21.0, 25.1)	22.9 (20.4, 25.4)	23.0 (20.7, 25.6)	22.6 (20.2, 24.8)	0.101
Female, n (%)	125 (70.6)	332 (66.1)	210 (67.7)	122 (63.5)	0.334
Etiology of PH					0.542
IPAH, n (%)	–	300 (59.8)	182 (58.7)	118 (61.5)	
CTEPH, n (%)	–	202 (40.2)	128 (41.3)	74 (38.5)	
WHO FC					**< 0.001**
I and II, n (%)	141 (79.7)	261 (52.0)	184 (59.4)	77 (40.1)	
III and IV, n (%)	36 (20.3)	241 (48.0)	126 (40.6)	115 (59.9)	
NT-proBNP, ng/L	117.2 (46.9, 325.0)	993.7 (287.0, 2095.5)**	752.2 (202.7, 1952.3)	1328.5 (568.0, 2374.0)	**< 0.001**
6MWD, m	462.1 ± 88.2	403.5 (330.0, 464.8)**	414.0 (335.3, 473.3)	394.0 (315.5, 446.5)	**0.044**
Newly diagnosed, n (%)	–	291 (58.0)	178 (57.4)	113 (58.9)	0.752
Pulmonary diseases, n (%)	17 (9.6)	43 (8.6)	22 (7.1)	21 (10.9)	0.135
Respiratory tract infection	5 (2.8)	16 (3.2)	9 (2.9)	7 (3.6)	
Bronchial asthma	3 (1.7)	3 (0.6)	1 (0.3)	2 (1)	
Emphysema	4 (2.3)	8 (1.6)	3 (1)	5 (2.6)	
Bronchiectasis	2 (1.1)	10 (2)	5 (1.6)	5 (2.6)	
Bronchitis	3 (1.7)	6 (1.2)	4 (1.3)	2 (1)	
PH specific therapy					0.962
None, n (%)	–	59 (11.8)	38 (12.3)	21 (10.9)	
Monotherapy, n (%)	–	315 (62.8)	192 (61.9)	123 (64.1)	
Double, n (%)	–	120 (23.9)	75 (24.2)	45 (23.4)	
Triple, n (%)	–	8 (1.6)	5 (1.6)	3 (1.6)	
Intervention^$^, n (%)	–	120 (23.9)	79 (25.5)	41 (21.4)	0.292

Data are presented as median (range) or number (percentage). CTEPH, chronic thromboembolic pulmonary hypertension; CW, clinical worsening; IPAH, idiopathic pulmonary arterial hypertension; NT-proBNP, N-terminal pro-brain natriuretic peptide; PH, pulmonary hypertension; 6MWD, 6-min walk distance; WHO FC, World Health Organization function class; ^$^ Intervention including pulmonary endarterectomy and balloon pulmonary angioplasty. *P < 0.05, Normal control compared with patients with PH. **P < 0.001, Normal control compared with patients with PH. ^#^Patients with clinical worsening compared with those without. Significant *P* values (*P *< 0.05) are bolded

**Table 2:** Echocardiographic, Hemodynamic and CPET-derived Parameters of Included Patients

Variable	Normal control (n = 177)	PH			*P* value^#^
		Total (n = 502)	Non-CW (n = 310)	CW (n = 192)	
Echocardiography					
LA, mm	33 (30, 37)	30 (28, 33)**	30 (28, 33)	30 (27, 33)	0.625
EF, %	65 (60, 68)	63.0 (60.0, 67.1)	63.5 (60.0, 67.2)	62.0 (60.0, 67.4)	0.231
RVED/LVED	0.56 (0.49, 0.66)	0.85 (0.68, 1.06)**	0.83 (0.67, 1.00)	0.91 (0.73, 1.10)	**0.001**
TRV, m/s	2.90 (2.65, 3.21	4.40 ± 0.60**	4.40 ± 0.61	4.40 ± 0.57	0.879
RHC					
S_v_O_2_, %	76.6 (73.2, 80.0)	69.2 ± 6.7**	70.2 ± 6.5	67.6 ± 6.6	**< 0.001**
mRAP, mmHg	3 (1, 5)	5 (2, 8)**	5.0 (2.0, 7.5)	5 (3, 8)	0.073
mPAP, mmHg	17 (14, 20)	51(44, 60)**	51 (43, 60)	52 (45, 62)	0.151
PAWP, mmHg	7 (2, 10)	7 (5, 10)	7.5 (5.0, 10.0)	7 (4, 10)	0.283
CI, L/min/m^2^	3.50 (2.96, 4.44)	2.82 (2.36, 3.39)**	2.87 (2.43, 3.48)	2.70 (2.26, 3.23)	**0.008**
PVR, wood units	1.66 (1.02, 2.47)	10.9 (7.8, 14.0)**	10.5 (7.4, 13.7)	11.7 (8.5, 14.7)	**0.003**
CPET					
FVC/prediction, %	90 ± 17	86 (76, 94)**	86 (77, 94)	85 (75, 95)	0.394
FEV_1_/prediction, %	83.1 ± 17.6	78 (69, 88)**	79 (69, 88)	77.5 (69.0, 87.0)	0.547
FEV_1_/FVC	0.79 (0.73, 0.84)	0.78 (0.73, 0.83)	0.78 (0.73, 0.83)	0.78 (0.73, 0.83)	0.375
Workload@Peak, watt	92 (71, 115)	66 (51, 87)**	67.0 (52.5, 90.5)	63.5 (49, 83)	0.141
VO_2_@Rest, mL/min/kg	4.69 (4.19, 5.20)	4.79 (4.20, 5.25)	4.79 (4.16, 5.30)	4.79 (4.30, 5.21)	0.774
VO_2_@Peak, mL/min/kg	18 ± 5	12.1 (10.2, 14.8)**	12.5 (10.4, 15.4)	11.5 (9.5, 14.1)	**0.003**
HR@Rest, beat/min	78 (68, 89)	81.0 (72.8, 90.0)	81.0 (72.8, 90.0)	80.5 (72.3, 90.8)	0.910
HR@Peak, beat/min	136.8 ± 24.2	137 (120, 154)	139 (121, 155)	134.0 (118.3, 153.0)	0.165
SBP@Rest, mmHg	113.4 ± 16.9	105.0 (96.0, 116.8)**	106 (96, 117)	104 (95, 114)	0.126
SBP@Peak, mmHg	147.8 ± 32.3	131 (111, 153)**	135 (114, 154)	127.0 (108.0, 149.8)	**0.030**
DBP@Rest, mmHg	77.5 (69.0, 90.3)	72 (65, 78)**	72 (65, 79)	71 (63, 78)	0.343
DBP@Peak, mmHg	85 (73.3, 102.8)	82 (70, 98)	82 (72, 99)	81.5 (69.0, 96.8)	0.381
VO_2_/HR@Rest, mL/beat	3.70 (3.0, 4.40)	3.60 (3.00, 4.30)	3.60 (3.00, 4.30)	3.55 (3.03, 4.20)	0.715
VO_2_/HR@Peak, mL/beat	8.00 (6.75, 9.35)	5.50 (4.60, 6.63)**	5.50 (4.80, 6.80)	5.40 (4.33, 6.40)	**0.016**
P_ET_CO_2_@Rest, mmHg	33 (31, 36)	27 (24, 30)**	27 (24, 30)	27 (24, 30)	0.430
P_ET_CO_2_@Peak, mmHg	37.0 (33.5, 41.0)	24 (19, 27)**	24 (20, 28)	22.5 (19.0, 26.0)	**0.001**
VE/VCO_2_ slope	33.3 (30.7, 36.5)	44.1 (37.9, 51.1)**	43.9 (37.2, 50.7)	45.2 (39.1, 52.9)	0.054
Ventilatory power, mmHg	4.66 (3.66, 5.49)	3.00 (2.25, 3.84)**	3.12 (2.33, 4.04)	2.73 (2.08, 3.58)	**0.002**

Data are presented as mean ± standard deviation, median (range) or number (percentage). @Rest, at rest; @Peak, at peak exercise; CI, cardiac index; CPET, cardiopulmonary exercise testing; CW, clinical worsening; DBP, diastolic blood pressure; EF, ejection fraction; FEV_1_, forced expiratory volume in one second; FVC, forced vital capacity; HR, heart rate; LA, anteroposterior diameter of left atrium; mPAP, mean pulmonary artery pressure; mRAP, mean right atrial pressure; PAWP, pulmonary artery wedge pressure; P_ET_CO_2_, end‐tidal partial pressure of carbon dioxide; PVR, pulmonary vascular resistance; RHC, right heart catheterization; RVED/LVED, the ratio of right ventricular end-diastolic to left ventricular end-diastolic diameter; SBP, systolic blood pressure; S_v_O_2_, mixed venous oxygen saturation; TRV, tricuspid regurgitation velocity; VCO_2_, carbon dioxide production; VE/VCO_2_, minute ventilation/carbon dioxide production; VO_2_, oxygen uptake; VO_2_, oxygen uptake; VO_2_/HR, oxygen consumption/ heart rate. *P < 0.05, Normal control compared with patients with PH. **P < 0.001, Normal control compared with patients with PH. ^#^ Patients with clinical worsening compared with those without. Significant *P* values (*P* < 0.05) are bolded

### Diagnostic performance of ventilatory power

**Fig. 2:**
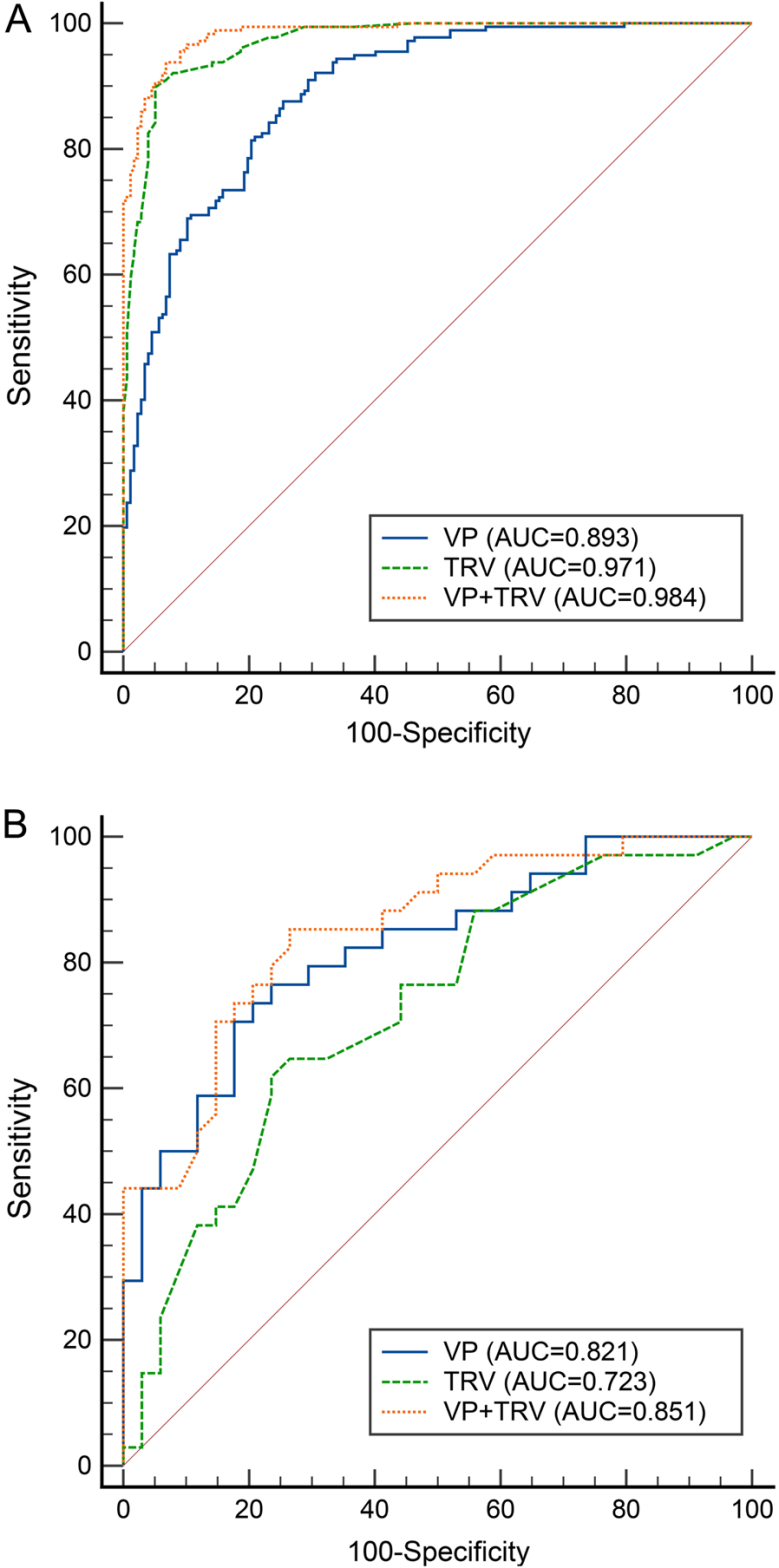
**A** ROC Curves of TRV and VP in Identifying Overt PH. TRV vs. VP, *P* < 0.001; TRV + VP vs. TRV, *P* = 0.002; TRV + VP vs. VP, *P* < 0.001. **B** ROC Curves of TRV and VP in Identifying Borderline PH. TRV vs. VP, *P* = 0.240; TRV + VP vs. TRV, *P* = 0.038; TRV + VP vs. VP, *P* = 0.331. PH, pulmonary hypertension; TRV, tricuspid regurgitation velocity; VP, ventilatory power

When differentiating patients with PH (mPAP ≥ 25 mmHg) from those without PH (mPAP < 25 mmHg), TRV was superior to ventilatory power [AUC: 0.971 (95%CI: 0.947–0.986) vs. 0.893 (95%CI:0.855–0.923), *P* < 0.001] (Fig. 2). The cutoff value, with the largest Youden-index, was 3.72 m/s for TRV (Sensitivity = 89.8%, Specificity = 94.9%, Positive predictive value = 94.6%, Negative predictive value = 90.3%, Accuracy = 92.4%) and 3.69 mmHg for ventilatory power (Sensitivity = 87.6%, Specificity = 74.6%, Positive predictive value = 77.5%, Negative predictive value = 85.7%, Accuracy = 81.1%). Meanwhile, the combination of these two variables (AUC: 0.984, 95%CI: 0.965–0.994; cutoff value = 0.43, Sensitivity = 93.8%, Specificity = 93.2%, Positive predictive value = 92.2%, Negative predictive value = 94%, Accuracy = 93.1%) demonstrated a superior AUC than either of these two variables used alone (*P* < 0.001, compared to ventilatory power; *P* = 0.002, compared to TRV). Similar results were also observed within both treatment naïve and treated subgroups (Additional file 1: Fig. S1). In sensitivity analysis, we only included patients without pulmonary diseases from both the control and PH group and the results remained unchanged (Additional file 1: Fig. S2).

When differentiating patients with mPAP = 21–24 mmHg from those with mPAP ≤ 20 mmHg, ventilatory power was superior to TRV, although the difference didn’t reach statistical significance [AUC: 0.821 (95%CI: 0.709–0.903) vs. 0.723 (95%CI: 0.601–0.824), *P* = 0.240] (Fig. 2). The cutoff value was 3.15 m/s for TRV (Sensitivity = 64.7%, Specificity = 73.5%, Positive predictive value = 71%, Negative predictive value = 67.6%, Accuracy = 69.1%) and 4.75 mmHg for ventilatory power (Sensitivity = 70.6%, Specificity = 82.4%, Positive predictive value = 80%, Negative predictive value = 73.7%, Accuracy = 76.5%). Meanwhile, the combination of TRV and ventilatory power (AUC: 0.851, 95%CI: 0.744–0.926; cutoff value = 0.5, Sensitivity = 85.3%, Specificity = 73.5%, Positive predictive value = 76.3%, Negative predictive value = 83.3%, Accuracy = 79.4%) demonstrated a superior AUC than TRV used alone (*P* = 0.038, compared to TRV; *P* = 0.331, compared to ventilatory power). Sensitivity analysis, in which only included patients without pulmonary diseases, yielded similar results (Additional file 1: Fig. S3).

### Correlation between ventilatory power and PH severity

**Fig. 3:**
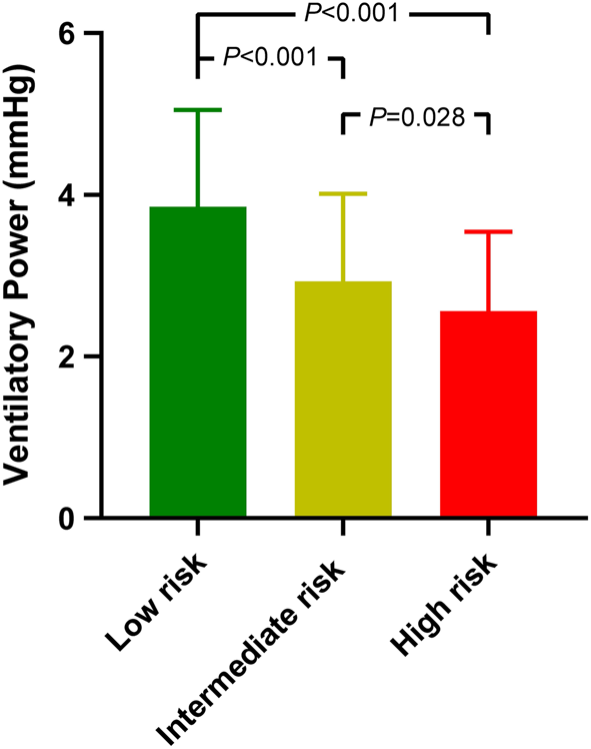
The Relationship between Ventilatory Power and the Abbreviated European Society of Cardiology /European Respiratory Society Risk Score. Data were present as mean ± SD

Among patients with PH (mPAP ≥ 25 mmHg), ventilatory power correlated with well-validated PH severity markers (Table 3). Moreover, the value of ventilatory power decreased as the abbreviated ESC/ERS risk score escalated. [low risk vs. intermediate risk vs. high risk: median (interquartile range), 3.73 (3.06, 4.57) mmHg vs. 2.72 (2.14, 3.56) mmHg vs. 2.17 (1.84, 3.09) mmHg, *P* < 0.001) (Fig. 3).

**Table 3:** Associations between Ventilatory Power and Parameters Reflecting PH Severity

Variable	Spearman coefficient	*P*-value
6MWD	0.406	**< 0.001**
NT-proBNP	− 0.419	**< 0.001**
TRV	− 0.207	**< 0.001**
RVED/LVED	− 0.348	**< 0.001**
S_v_O_2_	0.344	**< 0.001**
mPAP	− 0.247	**< 0.001**
CI	0.300	**< 0.001**
PVR	− 0.391	**< 0.001**
Workload@Peak	0.553	**< 0.001**
VO_2_@Peak	0.508	**< 0.001**
VO_2_/HR@Peak	0.392	**< 0.001**
SBP@Peak	0.719	**< 0.001**
DBP@Peak	0.438	**< 0.001**
P_ET_CO_2_@Peak	0.636	**< 0.001**
VE/VCO_2_ slope	− 0.686	**< 0.001**

@Peak, at peak exercise; CI, cardiac index; DBP, diastolic blood pressure; HR, heart rate; mPAP, mean pulmonary artery pressure; NT-proBNP, N-terminal pro-brain natriuretic peptide; PH, pulmonary hypertension; P_ET_CO_2_, end‐tidal partial pressure of carbon dioxide; PVR, pulmonary vascular resistance; RVED/LVED, the ratio of right ventricular end-diastolic to left ventricular end-diastolic diameter; SBP, systolic blood pressure; 6MWD, 6-min walk distance; SvO_2_, mixed venous oxygen saturation; TRV, tricuspid regurgitation velocity; VCO_2_, carbon dioxide production; VE/VCO_2_, minute ventilation/carbon dioxide production; VO_2_, oxygen uptake; VO_2_/HR, peak oxygen uptake/ heart rate. Significant *P* values (*P* < 0.05) are bolded

### Prognostic value of ventilatory power

Univariable Cox analysis identified that a number of demographics, hemodynamics and CPET-derived parameters had a *P* value < 0.100 (Table 4). The interaction between ventilatory power and etiology of PH was not statistically significant (*P* for interaction = 0.768), indicating that the predictive value of ventilatory power was comparable between CTEPH and IPAH. Similarly, no interaction was observed between ventilatory power and pulmonary diseases /PH treatment history (Table 4). Restricted cubic splines illustrated that the risk of clinical worsening decreased linearly as ventilatory power escalated (Additional file 1: Fig. S4). Then, variables with a *P* value < 0.100 in univariable Cox analysis were selected for multivariable Cox analysis with forward stepwise method (Likelihood Ratio). Results showed that only S_v_O_2_ and ventilatory power were independent predictors of clinical worsening (Table 4). The corrected C-index decreased in the following order: ventilatory power + the abbreviated ESC/ERS risk score (0.623) > ventilatory power + S_v_O_2_ (0.619) > ventilatory power (0.603) > the abbreviated ESC/ERS risk score (0.594).

**Table 4:** Univariable and Multivariable Cox Analysis for Long-term Clinical Worsening

Variable	Univariable model			Multivariable model		
	HR	95% CI	*P*-value	HR	95% CI	*P*-value
Age	1.004	0.995–1.014	0.337			
Body mass index	0.959	0.921–0.998	**0.041**			
Sex	0.954	0.710–1.281	0.754			
WHO FC	1.466	1.178–1.824	**0.001**			
NT-proBNP^$^	1.210	1.090–1.344	**< 0.001**			
6MWD	0.998	0.997–1.000	**0.014**			
PH specific therapy	1.215	0.972–1.519	0.088			
Intervention	1.310	0.922–1.861	0.132			
RVED/LVED	1.595	1.025–2.481	**0.039**			
EF	0.986	0.963–1.010	0.246			
TRV	0.998	0.781–1.276	0.990			
S_v_O_2_	0.959	0.941–0.979	**< 0.001**	0.955	0.932–0.980	**< 0.001**
mRAP	1.047	1.016–1.079	**0.003**			
mPAP	1.007	0.997–1.017	0.149			
CI	0.730	0.615–0.866	**< 0.001**			
PVR	1.046	1.019–1.074	**0.001**			
FVC	0.995	0.985–1.006	0.367			
FEV_1_	1.000	0.989–1.011	0.988			
FEV_1_/FVC	2.633	0.421–16.472	0.301			
Workload@Peak	0.993	0.987–0.998	**0.008**			
VO_2_@Rest	1.054	0.887–1.253	0.550			
VO_2_@Peak	0.941	0.904–0.980	**0.003**			
VO_2_/HR@Rest	0.927	0.788–1.090	0.359			
VO_2_/HR@Peak	0.891	0.815–0.975	**0.012**			
SBP@Rest	0.985	0.975–0.995	**0.002**			
SBP@Peak	0.994	0.989–0.998	**0.008**			
DBP@Rest	0.992	0.979–1.005	0.246			
DBP@Peak	0.995	0.989–1.001	0.108			
P_ET_CO_2_@Peak	0.962	0.937–0.987	**0.004**			
VE/VCO_2_ slope	1.017	1.004–1.030	**0.010**			
Ventilatory power	0.763	0.670–0.869	** < 0.001**	0.783	0.659–0.930	**0.005**
Etiology of PH	1.171	0.872–1.571	0.294			
Pulmonary diseases	1.118	0.710–1.761	0.629			
Newly diagnosed	0.831	0.621–1.112	0.214			
Ventilatory power × Etiology of PH*	0.879	0.373–2.073	0.768			
Ventilatory power × Pulmonary diseases*	0.995	0.945–1.048	0.854			
Ventilatory power × Newly diagnosed*	1.177	0.890–1.556	0.253			

@Rest, at rest; @Peak, at peak exercise; CI: cardiac index; DBP, diastolic blood pressure; EF, ejection fraction; FEV_1_: forced expiratory volume in one second; FVC: forced vital capacity; HR, heart rate; LA, anteroposterior diameter of left atrium; LV, left ventricular end-diastolic diameter; mRAP: mean right atrial pressure; mPAP: mean pulmonary artery pressure; NT-proBNP, N-terminal pro-brain natriuretic peptide; PAWP, pulmonary artery wedge pressure; PH, pulmonary hypertension; P_ET_CO_2_, end‐tidal partial pressure of carbon dioxide; PVR, pulmonary vascular resistance; RVED/LVED, the ratio of right ventricular end-diastolic to left ventricular end-diastolic diameter; SBP, systolic blood pressure; SvO_2_, mixed venous oxygen saturation; 6MWD, 6-min walk distance; TRV, tricuspid regurgitation velocity; VE/VCO_2_, minute ventilation/carbon dioxide production; VO_2_, oxygen uptake; VO_2_/HR, oxygen uptake / heart rate; WHO FC, World Health Organization function class; Etiology of PH is a dichotomic variable, which includes chronic thromboembolic pulmonary hypertension and idiopathic pulmonary arterial hypertension; ^$^for each increase of 1000 ng/L in NT-proBNP. *P for interaction. Significant *P *values (*P* < 0.05) are bolded

**Fig. 4:**
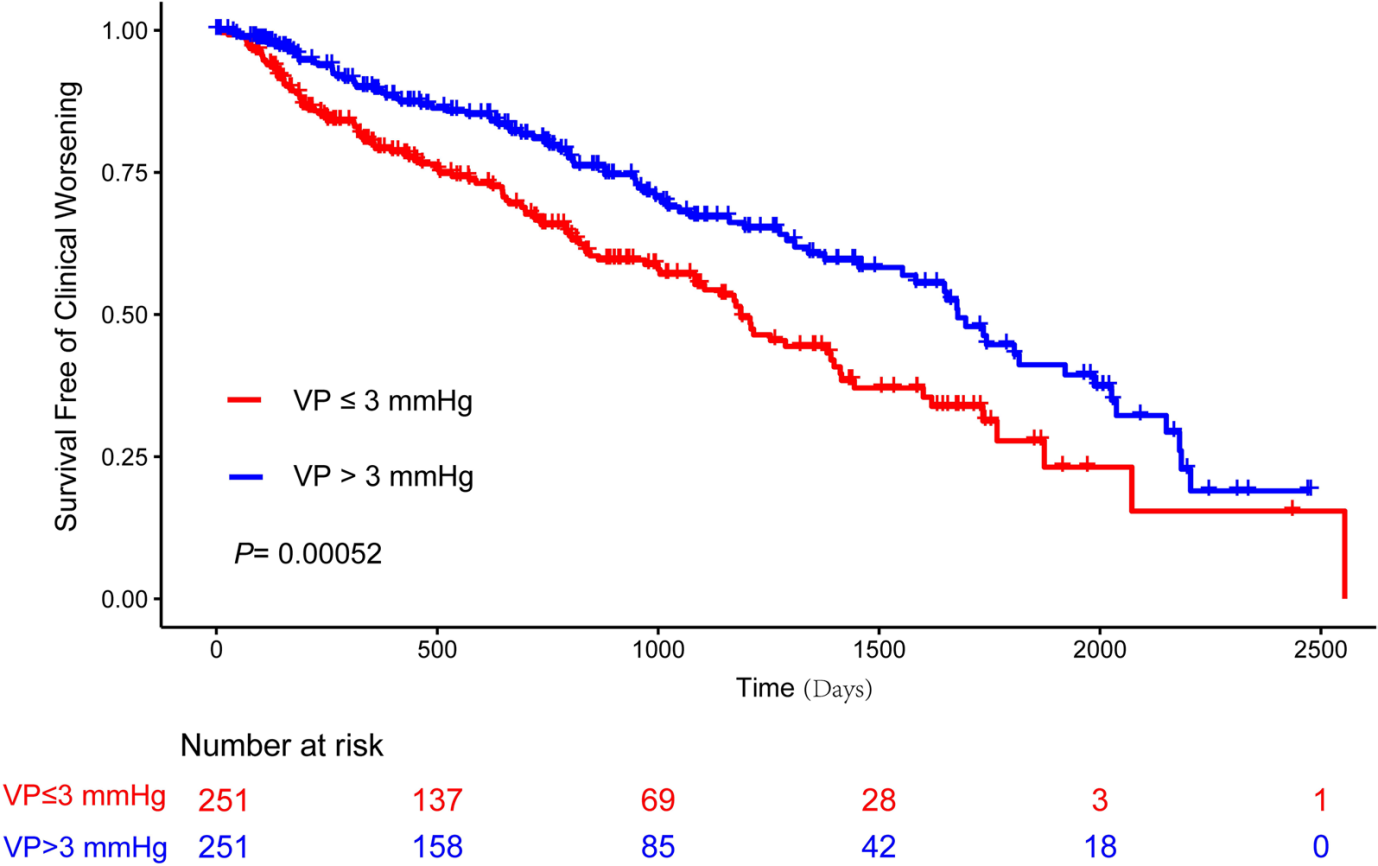
Kaplan–Meier Curve of VP in Predicting Clinical Worsening Events. VP, ventilatory power

According to median value of ventilatory power (3 mmHg), patients were further dichotomized into two groups, namely ventilatory power ≤ 3 mmHg and ventilatory power > 3 mmHg. Compared with patients with ventilatory power > 3 mmHg, patients with ventilatory power ≤ 3 mmHg had significantly poorer survival and shorter time to clinical worsening (Fig. 4).

### The impact of treatment strategy on ventilatory power at follow-up

**Fig. 5:**
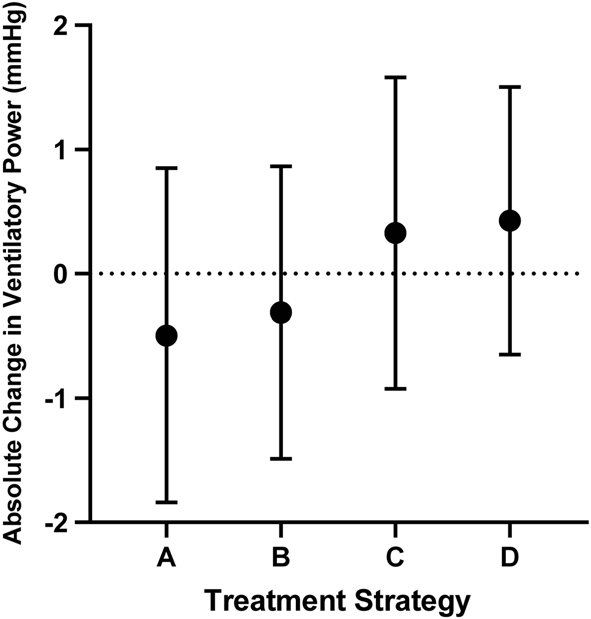
The Absolute Change of Ventilatory Power from Baseline to Last follow-up, Stratified by Treatment Strategy. **A** Unchanged vasodilators monotherapy or escalating to vasodilators monotherapy from treatment naïve (n = 53). **B** Escalating to combination PH medicine (n = 44). **C** Initial combination PH medicine (n = 38). **D** Undergoing pulmonary endarterectomy/balloon pulmonary angioplasty at baseline or follow-up (n = 81). Data were present as mean ± SD

Two hundred and sixteen patients had follow-up ventilatory power. The median time from baseline to the last follow-up ventilatory power was 24 (13, 39) months. Based on the treatment at baseline and follow-up, we categorized these patients into four groups. As shown in Fig. 5, the absolute change of ventilatory power from baseline to last follow-up declined in the following order: undergoing pulmonary endarterectomy/balloon pulmonary angioplasty > initial combination PH medicine > escalating to combination PH medicine > unchanged vasodilators monotherapy or escalating to vasodilators monotherapy from treatment naive.

## Discussion

Ventilatory power is a novel index reflecting both ventilation efficiency and peripheral blood flow. However, literature on this topic is scarce. To the best of our knowledge, the current study demonstrated for the first time that ventilatory power could improve the predictive power of TRV in identifying overt PH and borderline PH; ventilatory power was associated with well-validated markers of PH severity; ventilatory power was an independent predictor of clinical worsening in PH, and it could improve the predictive ability of the abbreviated ESC/ERS score.

In the present study, we observed that patients with PH had much lower ventilatory power than the control group. The underlying physiological mechanisms might be as follows. On one hand, patients with PH is featured by exaggerated ventilatory response to exercise [12]. Secondary to the elevated PVR and mPAP, right ventricle fails to increase cardiac output proportionally to elevated workload during exercise, leading to insufficient pulmonary perfusion. Moreover, blood flow accelerates during exercise, reducing time for internal respiration. Additionally, more blood flows to dead space during exercise, further compromising oxygen saturation. To compensate for insufficient pulmonary perfusion and compromised oxygen saturation, ventilation must increase out of proportion to carbon dioxide output to meet metabolic need, resulting in elevated VE/VCO_2_ slope. On another hand, patients with PH suffer from more blunted hemodynamic increment to exercise secondary to enlarged right ventricle, displaced septal and compressed left ventricular, which results in reduced SBP@Peak. Taken together, patients with PH had significantly lower ventilatory power than normal control, which was more profound in patients with clinical worsening.

In a retrospective study with 47 patients, Correale et al. [5] reported that ventilatory power could differentiate patients with overt PH from those with mPAP < 25 mmHg, which was consistent with our results. Additionally, we found ventilatory power could slightly improve the predictive power of TRV in identifying overt PH. Based on robust evidence, Simonneau et al. proposed revising the definition of pre-capillary PH as mPAP > 20 mmHg during the 6th World Symposium on PH [13]. We found that ventilatory power could discriminate borderline PH with high accuracy and combining ventilatory power and TRV could further improve the predictive ability.

We found that ventilatory power was associated with hemodynamics measured by RHC, which was consistent with the study by Correale et al. [5]. Additionally, we also found ventilatory power was also correlated with well-validated variables that reflected severity of PH, such as 6MWD, NT-proBNP, RVED/LVED, and VO_2_@Peak. More importantly, the value of ventilatory power decreased as the abbreviated ESC/ERS risk score escalated. Previous studies showed that ventilatory power was associated with disease severity in coronary artery disease [3] and left heart failure [14]. Hirashiki et al. [11] reported that, among various CPET-derived variables, ventilatory power was the only one significantly improved after 6 months PH treatment compared with 3 months. Going further, the present study showed that the more aggressive the treatment strategy was, the more profound ventilatory power was improved. Together with previous studies, our results suggested that ventilatory power may be a useful tool in assessing disease severity and monitoring response to PH treatment.

Previous studies have found that ventilatory power could independently predict prognosis of patients with left heart failure, and each unit increase in ventilatory power could reduce the risk of clinical worsening by 43% [4]. Current study extends the knowledge to patients with PH characterized by right heart failure. We found that each unit increase in ventilatory power could reduce the risk of clinical worsening by 22%. Moreover, ventilatory power could improve the predictive power of the abbreviated ESC/ERS risk score.

### Limitation

The current study had several limitations. First, only patients with IPAH and CTEPH were included in the current study, which limits the extrapolation of our conclusion. Second, a small number of patients had comorbid pulmonary diseases, which might have negative impact on the PH classification and ventilation efficiency. When pulmonary diseases co-exist, the classification of group 1 and 4 PH becomes very tricky. As a tertiary PH center in a cardiology specialized hospital, patients with severe pulmonary diseases and group 3 PH do not usually visit our clinic. This was the reason why only small number of patients had comorbid pulmonary diseases in the present study. Moreover, we do not usually categorize PH patients with confirmed COPD or other severe pulmonary diseases as idiopathic pulmonary arterial hypertension. Furthermore, in the present study, comorbid pulmonary diseases were mild, as evidenced by the fact that the FEV_1_/FVC was 0.78 (0.73, 0.83) for all included patients with PH, 0.78 (0.71, 0.82) for patients with PH and pulmonary diseases, and 0.79 (0.73, 0.83) for patients with PH but without pulmonary diseases. Usually, the mean pulmonary arterial pressure (mPAP) of the pure group 3 PH would not exceed 40 mmHg [7, 8]. To avoid controversies, patients with comorbid pulmonary diseases and mPAP ≤ 40 mmHg (n = 9) in this study were still excluded, while patients with comorbid pulmonary diseases and mPAP > 40 mmHg (n = 43) were considered group 1 or 4 PH dominant and were preserved. We also performed a sensitivity analysis within patients without pulmonary diseases, and the results were in line with the main analysis (Table 4, the P value for the interaction between ventilatory power and pulmonary diseases > 0.05; Additional file 1: Fig. S2 and S3; Additional file 2: Table S2). Third, as a tertiary PH center, patients with borderline PH were not commonly seen in our medical practice. Thus, the sample size was relatively small when evaluating the diagnostic performance of ventilatory power in identifying borderline PH. Our results need to be verified in the future studies with a prospective design and larger sample size.

## Conclusion

Ventilatory power could improve the predictive power of TRV in identifying overt PH and borderline PH. Moreover, ventilatory power could reflect disease severity and treatment response, independently predict clinical worsening events and improve the predictive power of the abbreviated ESC/ERS risk score.

## Supplementary Information

**Additional file 1:** Supplementary figures.

**Additional file 2:** Supplementary tables.

## Data Availability

The datasets used and/or analysed during the current study are available from the corresponding author on reasonable request.

## Abbreviations

AUC: Area under curve
CPET: Cardiopulmonary exercise test
CTEPH: Chronic thromboembolic pulmonary hypertension
ESC/ERS: The European Society of Cardiology /European Respiratory Society
IPAH: Idiopathic pulmonary arterial hypertension
mPAP: Mean pulmonary arterial pressure
NT-proBNP: N-terminal pro-B-type natriuretic peptide
PH: Pulmonary hypertension
VE/VCO_2_: Minute ventilation/ carbon dioxide production
ROC: Receiver operating characteristic
RVED/LVED: Right ventricular end-diastolic diameter/ left ventricular end-diastolic diameter
SBP@Peak: Systolic blood pressure at peak exercise
6-MWD: Six-minute walk distance
TRV: Tricuspid regurgitation velocity
VO_2_@Peak: Oxygen consumption at peak exercise
WHO FC: World Health Organization Function Class
