# Supplementary material for: Diagnostic and prognostic value of ventilatory power in pulmonary hypertension
Source: Respir Res. 2022 Oct 16;23:285. doi: 10.1186/s12931-022-02212-5 (PMC9575278; doi:10.1186/s12931-022-02212-5)
Supplement: Supplementary file 1 — Additional file 1: Supplementary figures. [file 12931_2022_2212_MOESM1_ESM.docx]

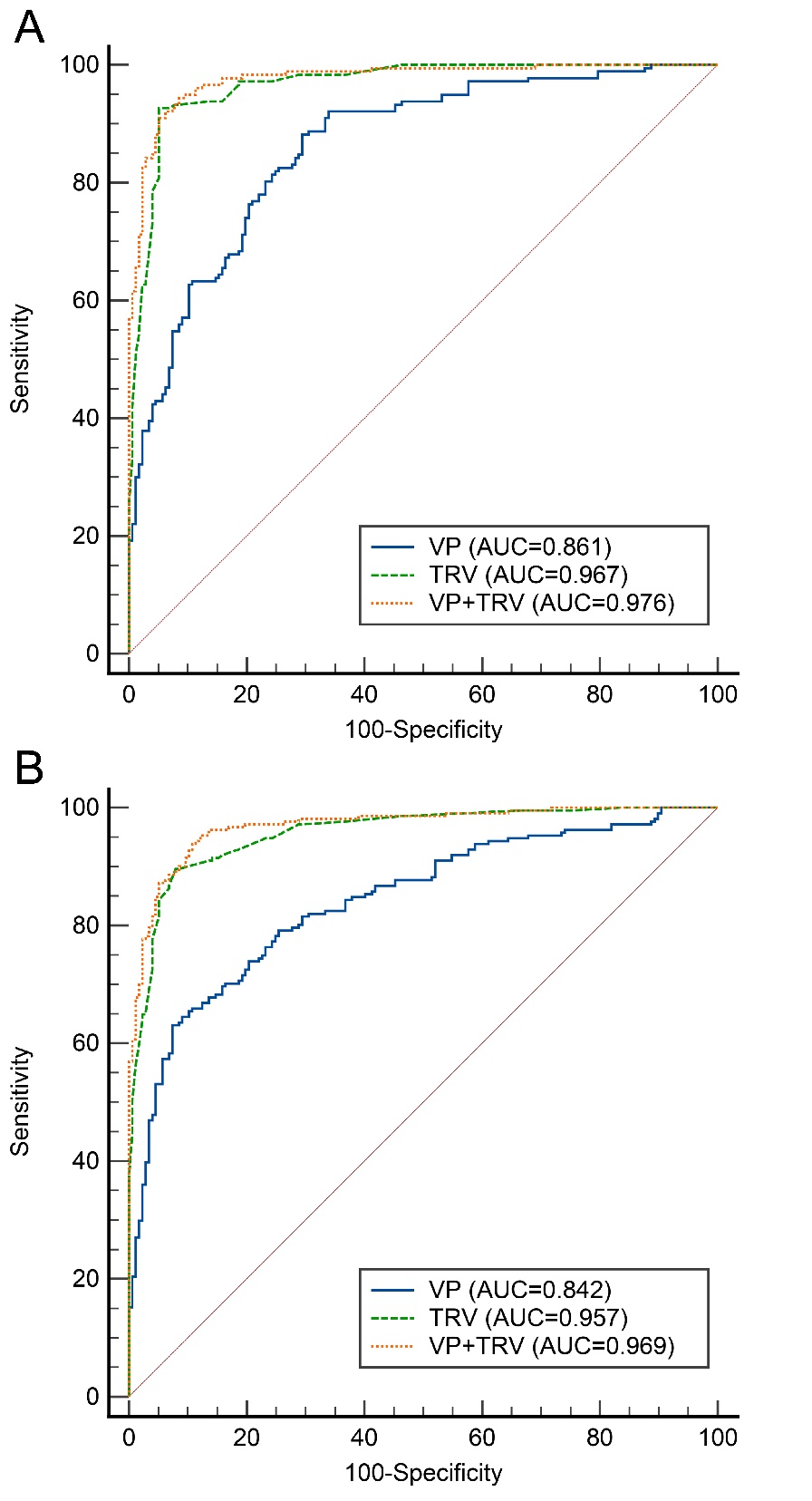


Figure S1. Subgroup Analysis of ROC Curves of TRV and VP in Identifying Overt PH. (A) treatment naïve patients: TRV (cutoff value=3.72, Sensitivity=92.7%, Specificity=94.9%, Positive predictive value=94.8%, Negative predictive value=92.8%, Accuracy=93.8%), VP (cutoff value=3.91, Sensitivity=88.1%, Specificity=70.6%, Positive predictive value=74.6%, Negative predictive value=85.6%, Accuracy=79.2%); VP+TRV (cutoff value=0.42, Sensitivity=94.4%, Specificity=91.5%, Positive predictive value=91.8%, Negative predictive value=94.2%, Accuracy=92.9%); TRV vs. VP, *P*<0.001; TRV+VP vs. TRV, *P*=0.030; TRV+VP vs. VP, *P*<0.001. (B) treated patients: TRV (cutoff value=3.58, Sensitivity=89.6%, Specificity=92.1%, Positive predictive value=93.1%, Negative predictive value=88.1%, Accuracy=90.7%), VP (cutoff value=2.96, Sensitivity=63%, Specificity=92.7%, Positive predictive value=91.1%, Negative predictive value=67.8%, Accuracy=76.5%); VP+TRV (cutoff value=0.36, Sensitivity=93.8%, Specificity=89.3%, Positive predictive value=91.2%, Negative predictive value=92.4%, Accuracy=91.8%); TRV vs. VP, P<0.001; TRV+VP vs. TRV, P=0.017; TRV+VP vs. VP, P<0.001.


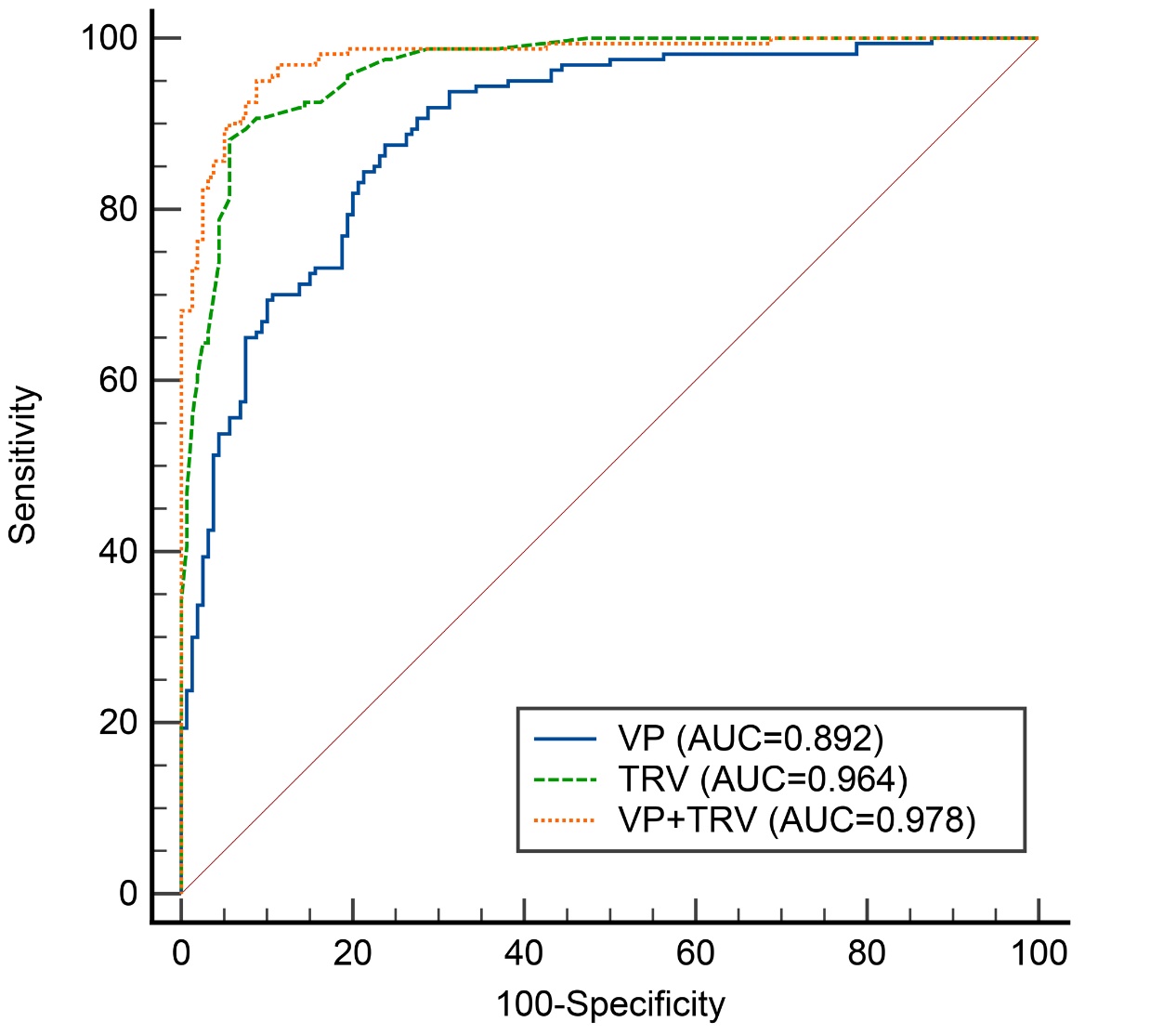
Figure S2. Sensitivity Analysis of ROC Curves of TRV and VP in Identifying Overt PH. Only including patients without pulmonary diseases from the control and PH group. TRV (cutoff value=3.72, Sensitivity=88.1%, Specificity=94.4%, Positive predictive value=94%, Negative predictive value=88.8%, Accuracy=91.3%), VP (cutoff value=3.67, Sensitivity=86.3%, Specificity=76.3%, Positive predictive value=78.4%, Negative predictive value=84.7%, Accuracy=81.3%); VP+TRV (cutoff value=0.35, Sensitivity=95%, Specificity=91.2%, Positive predictive value=91.6%, Negative predictive value=94.8%, Accuracy=93.1%); TRV vs. VP, *P*<0.001; TRV+VP vs. TRV, *P*=0.008; TRV+VP vs. VP, *P*<0.001.


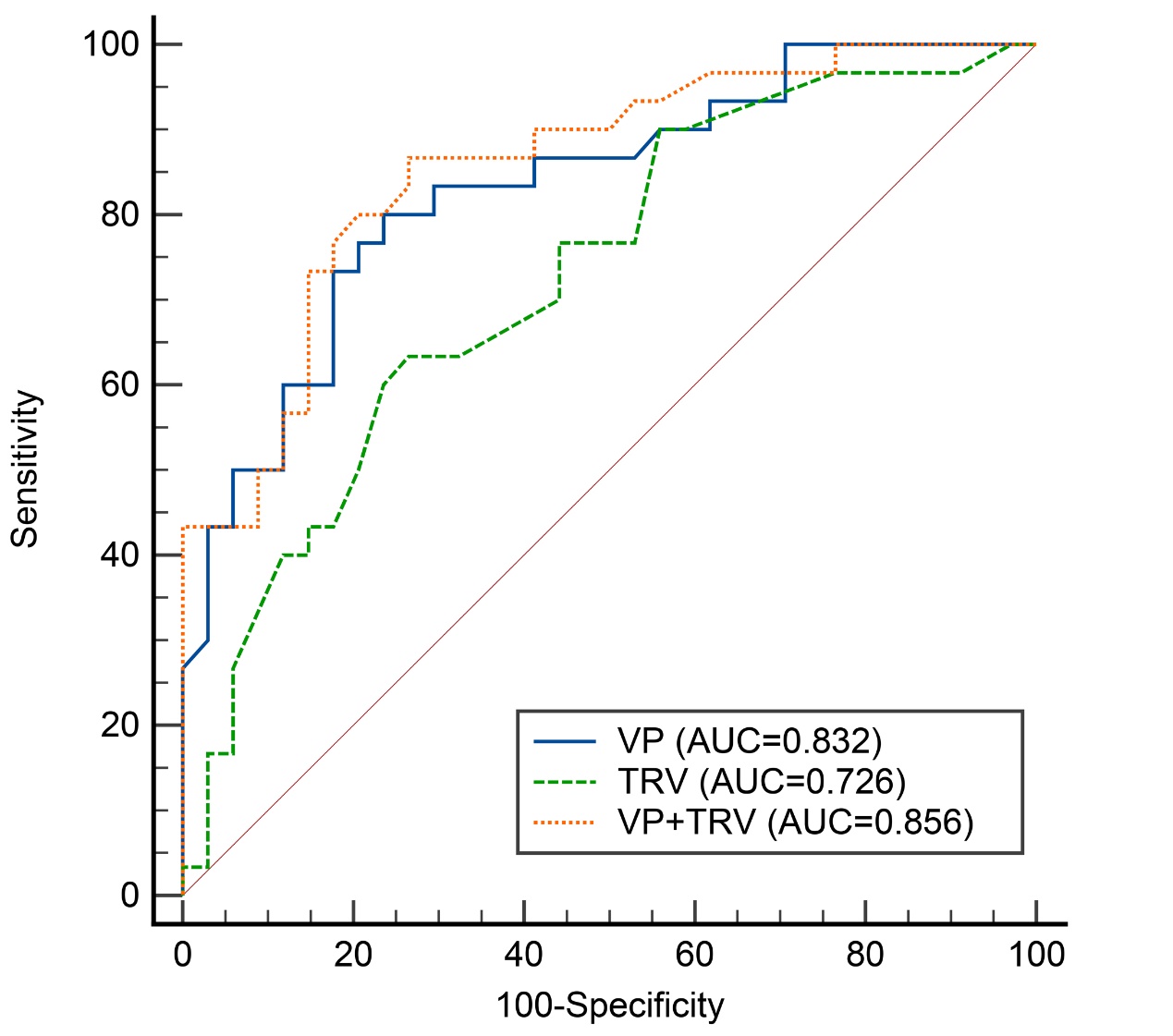


Figure S3. Sensitivity Analysis of ROC Curves of TRV and VP in Identifying borderline PH. Only including patients without pulmonary diseases from the control and PH group. TRV (cutoff value=3.15, Sensitivity=63.3%, Specificity=73.5%, Positive predictive value=67.9%, Negative predictive value=69.4%, Accuracy=68.8%), VP (cutoff value=4.94, Sensitivity=80%, Specificity=76.5%, Positive predictive value=75%, Negative predictive value=81.3%, Accuracy=78.1%); VP+TRV (cutoff value=0.46, Sensitivity=86.7%, Specificity=73.5%, Positive predictive value=91.6%, Negative predictive value=94.8%, Accuracy=93.1%); TRV vs. VP, *P*=0.210; TRV+VP vs. TRV, *P*=0.040; TRV+VP vs. VP, *P*=0.449.


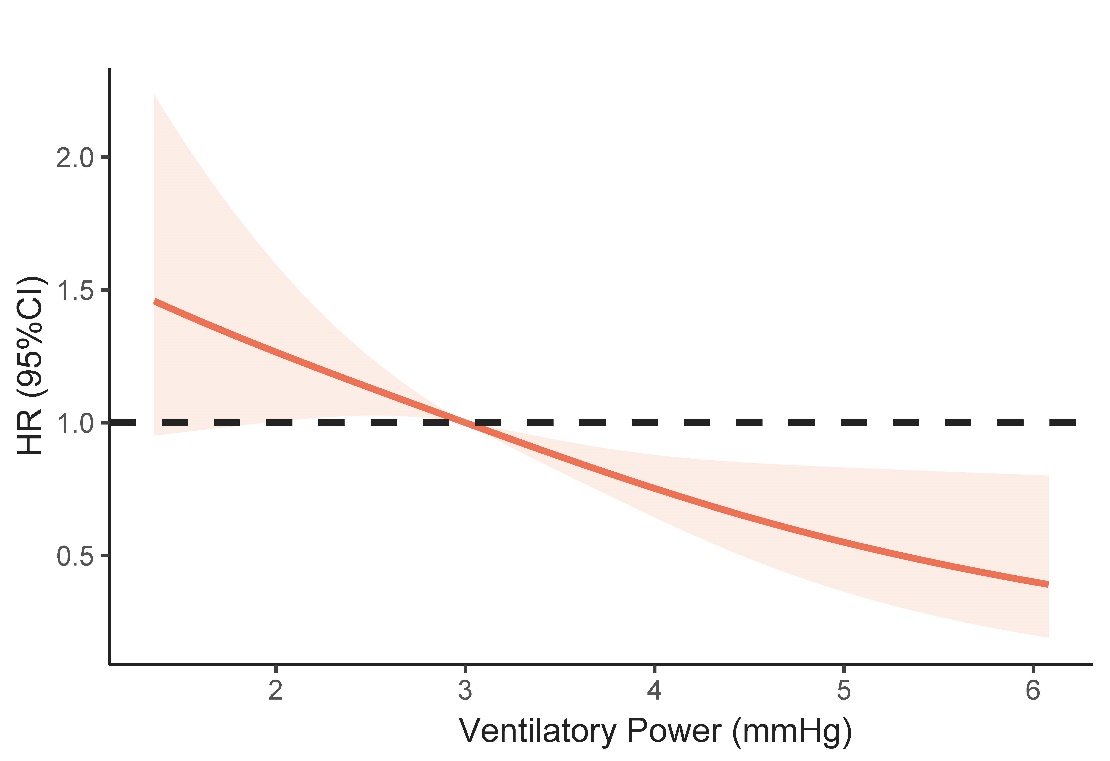


Figure S4. Restricted Cubic Splines of Ventilatory Power. CI, confidence interval; HR, hazard ratio.
