# Supplementary material for: Diagnostic and prognostic value of ventilatory power in pulmonary hypertension
Source: Respir Res. 2022 Oct 16;23:285. doi: 10.1186/s12931-022-02212-5 (PMC9575278; doi:10.1186/s12931-022-02212-5)
Supplement: Supplementary file 2 — Additional file 2: Supplementary tables. [file 12931_2022_2212_MOESM2_ESM.docx]

Table S1 The Scoring of the Abbreviated ESC/ERS Risk Score.

| Points assigned | 1 | 2 | 3 |
| --- | --- | --- | --- |
| WHO FC | I/II | III | IV |
| 6MWD, m | >440 | 165-440 | <165 |
| BNP, ng/L  Or  NT-proBNP, ng/L | <50  Or  <300 | 50-300  Or  300-1400 | >300  Or  >1400 |
| Right atrial pressure, mmHg | <8 | 8-14 | >14 |
| Cardiac index, L/min/m^2^ | ≥2.5 | 2.0-2.4 | <2.0 |
| S_v_O_2_, % | >65 | 60-65 | <60 |

BNP, brain natriuretic peptide; ESC/ERS, the European Society of Cardiology /European Respiratory Society; NT-proBNP, N-terminal pro-brain natriuretic peptide; 6MWD, 6-minute walk distance; S_v_O_2_: mixed venous oxygen saturation; WHO-FC, world Health Organization function class.

Table S2 Univariate and Multivariate Cox Analysis for Long-Term Clinical Worsening^#^

| Variable | Univariate model | | | Multivariate model | | |
| --- | --- | --- | --- | --- | --- | --- |
|  | HR | 95% CI | *P*-value | HR | 95% CI | *P*-value |
| Age | 1.003 | 0.993-1.013 | 0.506 |  |  |  |
| Body mass index | 0.959 | 0.919-1.001 | **0.056** |  |  |  |
| Sex | 1.019 | 0.742-1.400 | 0.908 |  |  |  |
| WHO FC | 1.585 | 1.259-1.994 | **<0.001** |  |  |  |
| NT-proBNP^$^ | 1.242 | 1.110-1.390 | **<0.001** |  |  |  |
| 6MWD | 0.998 | 0.996-0.999 | **0.003** |  |  |  |
| PH specific therapy | 1.211 | 0.961-1.526 | 0.104 |  |  |  |
| Intervention | 1.269 | 0.880-1.831 | 0.202 |  |  |  |
| RVED/LVED | 2.115 | 1.293-3.560 | **0.003** |  |  |  |
| EF | 0.986 | 0.961-1.012 | 0.304 |  |  |  |
| TRV | 1.071 | 0.828-1.386 | 0.599 |  |  |  |
| S_v_O_2_ | 0.961 | 0.940-0.981 | **<0.001** | 0.960 | 0.934-0.986 | **0.003** |
| mRAP | 1.039 | 1.004-1.076 | **0.030** |  |  |  |
| mPAP | 1.007 | 0.997-1.018 | 0.161 |  |  |  |
| CI | 0.719 | 0.599-0.864 | **<0.001** |  |  |  |
| PVR | 1.050 | 1.020-1.081 | **0.001** |  |  |  |
| FVC | 0.991 | 0.980-1.003 | 0.150 |  |  |  |
| FEV_1_ | 0.997 | 0.986-1.009 | 0.668 |  |  |  |
| FEV1/FVC | 3.558 | 0.513-24.679 | 0.199 |  |  |  |
| Workload@Peak | 0.991 | 0.985-0.997 | **0.002** |  |  |  |
| VO_2_@Rest | 1.028 | 0.853-1.239 | 0.771 |  |  |  |
| VO_2_@Peak | 0.934 | 0.895-0.974 | **0.001** |  |  |  |
| VO_2_/HR@Rest | 0.895 | 0.754-1.063 | 0.206 |  |  |  |
| VO_2_/HR@Peak | 0.882 | 0.803-0.969 | **0.009** |  |  |  |
| SBP@Rest | 0.984 | 0.974-0.994 | **0.002** |  |  |  |
| SBP@Peak | 0.993 | 0.989-0.998 | **0.008** |  |  |  |
| DBP@Rest | 0.989 | 0.975-1.003 | 0.110 |  |  |  |
| DBP@Peak | 0.994 | 0.987-1.000 | **0.061** |  |  |  |
| P_ET_CO_2_@Peak | 0.963 | 0.937-0.991 | **0.009** |  |  |  |
| VE/VCO_2_ slope | 1.017 | 1.003-1.031 | **0.019** |  |  |  |
| Ventilatory power | 0.769 | 0.671-0.881 | **<0.001** | 0.781 | 0.653-0.935 | **0.007** |
| Etiology of PH | 1.147 | 0.841-1.565 | 0.386 |  |  |  |
| Newly diagnosed | 0.809 | 0.595-1.101 | 0.178 |  |  |  |
| Ventilatory power * Etiology of PH | 1.033 | 0.768-1.390 | 0.829 |  |  |  |
| Ventilatory power * Newly diagnosed | 1.135 | 0.852-1.512 | 0.386 |  |  |  |

@Rest, at rest; @Peak, at peak exercise; CI: cardiac index; DBP, diastolic blood pressure; EF, ejection fraction; FEV_1_: forced expiratory volume in one second; FVC: forced vital capacity; HR, heart rate; LA, anteroposterior diameter of left atrium; LV, left ventricular end-diastolic diameter; mRAP: mean right atrial pressure; mPAP: mean pulmonary artery pressure; NT-proBNP, N-terminal pro-brain natriuretic peptide; PAWP, pulmonary artery wedge pressure; PH, pulmonary hypertension; P_ET_CO_2_, end‐tidal partial pressure of carbon dioxide; PVR, pulmonary vascular resistance; RVED/LVED, the ratio of right ventricular end-diastolic to left ventricular end-diastolic diameter; SBP, systolic blood pressure; SvO_2_, mixed venous oxygen saturation; 6MWD, 6-minute walk distance; TRV, tricuspid regurgitation velocity; VE/VCO_2_, minute ventilation/carbon dioxide production; VO_2_, oxygen uptake; VO_2_/HR, oxygen uptake / heart rate; WHO FC, World Health Organization function class; Etiology of PH is a dichotomic variable, which includes chronic thromboembolic pulmonary hypertension and idiopathic pulmonary arterial hypertension; ^#^sensitivity analysis performed in patients without respiratory impairment ^$^for each increase of 1000 ng/L in NT-proBNP. * *P* for interaction
